# Molecular evidence and clinical importance of β‐arrestins expression in patients with acromegaly

**DOI:** 10.1111/jcmm.13427

**Published:** 2018-01-27

**Authors:** Maria Caroline Alves Coelho, Marina Lipkin Vasquez, Luiz Eduardo Wildemberg, Mari C. Vázquez‐Borrego, Luciana Bitana, Aline Helen da Silva Camacho, Débora Silva, Liana Lumi Ogino, Nina Ventura, Leila Chimelli, Raul M. Luque, Leandro Kasuki, Mônica R. Gadelha

**Affiliations:** ^1^ Neuroendocrinology Research Center/Endocrinology Division Medical School and Hospital Universitário Clementino Fraga Filho Universidade Federal do Rio de Janeiro Rio de Janeiro Brazil; ^2^ Endocrine Division Hospital Universitário Pedro Ernesto Universidade Estadual do Rio de Janeiro Rio de Janeiro Brazil; ^3^ Endocrine Division Instituto Estadual de Diabetes e Endocrinologia Luiz Capriglione Rio de Janeiro Brazil; ^4^ Molecular Genetics Laboratory Instituto Estadual do Cérebro Paulo Niemeyer Rio de Janeiro Brazil; ^5^ Neuroendocrinology Division Instituto Estadual do Cérebro Paulo Niemeyer Rio de Janeiro Brazil; ^6^ Maimonides Institute for Biomedical Research of Cordoba (IMIBIC) Córdoba Spain; ^7^ Department of Cell Biology, Physiology, and Immunology Universidad de Córdoba Córdoba Spain; ^8^ Reina Sofia University Hospital Córdoba Spain; ^9^ CIBER Fisiopatología de la Obesidad y Nutrición (CIBERobn) Córdoba Spain; ^10^ Neuropathology Laboratory Instituto Estadual do Cérebro Paulo Niemeyer Rio de Janeiro Brazil; ^11^ Pathology Division Instituto Nacional do Câncer Rio de janeiro Brazil; ^12^ Radiology Division Instituto Estadual do Cérebro Paulo Niemeyer Rio de Janeiro Brazil; ^13^ Endocrine Division Hospital Federal de Bonsucesso Rio de Janeiro Brazil

**Keywords:** acromegaly, β‐arrestins, somatostatin receptors, dopamine receptor, somatostatin receptor ligands

## Abstract

β‐arrestins seem to have a role in endocytosis and desensitization of somatostatin receptor subtype 2 (sst2) and could be associated with the responsiveness to somatostatin receptor ligands (SRL) in patients with acromegaly. To investigate the *in vivo* correlation between β‐arrestins 1 and 2 with sst2*,* sst5 and dopamine receptor subtype 2 (D2) expressions, and the association of β‐arrestins with response to first‐generation SRL and invasiveness in somatotropinomas. *β‐arrestins 1* and *2*,* sst2*,* sst5* and *D2* mRNA expressions were evaluated by quantitative real‐time RT‐PCR on tumoral tissue of 96 patients. Moreover, sst2 and sst5 protein expressions were also evaluated in 40 somatotropinomas by immunohistochemistry. Response to SRL, defined as GH <1 μg/l and normal IGF‐I levels, was assessed in 40 patients. The Knosp‐Steiner criteria were used to define invasiveness. Median *β‐arrestin 1*,* β‐arrestin* 2, *sst2*,* sst5* and *D2* mRNA copy numbers were 478; 9375; 731; 156**;** and 3989, respectively. There was a positive correlation between *β‐arrestins 1* and *2* (*R *=* *0.444, *P *<* *0.001). However, no correlation between *β‐arrestins* and sst2, sst5 (mRNA and protein levels) or *D2* was found. No association was found between *β‐arrestins* expression and SRL responsiveness or tumour invasiveness. Although previous data suggest a putative correlation between β‐arrestins and sst2, our data clearly indicated that no association existed between β‐arrestins and sst2, sst5 or D2 expression, nor with response to SRL or tumour invasiveness. Therefore, further studies are required to clarify whether β‐arrestins have a role in the response to treatment with SRL in acromegaly.

## Introduction

Acromegaly is a chronic disease due to hypersecretion of growth hormone (GH), commonly caused by a GH‐secreting pituitary adenoma (somatotropinoma). Surgery is the first‐line treatment; however, approximately half of the patients will not be cured, especially those with macroadenomas. In such cases, medical adjuvant treatment is often necessary, and the first‐generation somatostatin receptor ligands (SRL) are the first choice 1.

The first‐generation SRL, octreotide and lanreotide, act mainly through somatostatin receptor subtype 2 (sst2), and the expression of this receptor is used as a predictor of response to SRL 2, 3. However, there are patients who, despite high sst2 expression levels, do not respond to SRL therapy 4. Several studies have been conducted with the aim of identifying the molecular basis of partial or total resistance to these drugs, varying from reduced density of the sst subtypes 2, receptors mutation 5 to changes in signal transduction and cytoskeleton proteins involvement, *etc*. 6. The recognition of a subset of patients unlikely to respond to SRL therapy might allow the rational use of these drugs in patients with acromegaly 7.

In this sense, it was recently described that the expression of β‐arrestins could lead to changes in the signalling pathway of sst2 in somatotropinomas and consequently could be related to the responsiveness to first‐generation SRL 6, 8. Specifically, β‐arrestins bind to G protein‐coupled receptors (GPCRs) after their phosphorylation by the G protein‐coupled receptor kinase (GRK) type 2, allowing the recruitment of proteins, regulating the trafficking of proteins and the endocytic machinery. After stabilization of binding with GPCR, β‐arrestins undergo conformational change that allows their connection with the endocytic machinery dependent on clathrin 9, allowing the internalization of β‐arrestins‐GPCR complex, leading to receptor desensitization 9. The stability of the arrestin‐receptor complex appears to be determinant for the recycling rate and internalization of GPCRs. Indeed, this process of arrestin‐induced endocytosis of the sst2 was observed in cell cultures 10.

β‐arrestins are considered multifunctional adaptor molecules, with several binding partners. *In vitro* study showed that β‐arrestins regulate the activation of multiple components of MAPK cascade, such as ERK, redirecting these components from the nucleus to the cytoplasm and resulting in a decrease in proliferation 11. In fact, changes in β‐arrestins expression (at the mRNA and protein level) have been associated with more aggressive cancer phenotypes 12, 13, 14.

Based on the data reported to date, the aim of this study was to further investigate the putative *in vivo* correlation between the expression of *β‐arrestins 1* and *2* with sst2*,* sst5 or *dopamine receptor type 2* (D2) and also the association between *β‐arrestins* expression with the response to first‐generation SRL and with the presence of cavernous sinus invasion in somatotropinomas.

## Subjects and methods

This study was approved by the Ethics Committee of Hospital Universitário Clementino Fraga Filho and Medical School/Universidade Federal do Rio de Janeiro. All patients signed an informed consent before entering the study.

### Patients and tumours

Consecutive patients with acromegaly who had fresh tumour samples available obtained during the transsphenoidal surgery performed at the Instituto Estadual do Cérebro Paulo Niemeyer and at the Hospital Universitário Clementino Fraga Filho were included in this study. The tumour was immediately snap‐frozen and stored at −80°C for molecular biology studies, and available formalin fixed paraffin‐embedded tissues were used for immunohistochemistry.

The diagnosis of acromegaly was performed according to current criteria 1. The maximum diameter of the tumour and the presence or absence of cavernous sinus invasion, using the modified Knosp‐Steiner criteria 15, were evaluated by the same radiologist analysing the preoperative magnetic resonance image (MRI).

#### Criteria for cure and response to first‐generation SRL

Patients with nadir GH levels after oral glucose tolerance test (OGTT) higher than 1.0 ng/ml or age‐matched plasma high IGF‐I levels three months after surgery were considered not cured. Subsequently, the biochemical response to medical treatment was evaluated by GH and IGF‐I levels after 6 months of treatment with octreotide LAR at maximum dose of 30 mg or lanreotide autogel at maximum dose of 120 mg. Uncontrolled patients were those who had GH levels >1.0 μg/l and/or IGF‐I levels higher than age‐matched normal patients.

### Methods

#### Hormonal assessment

GH was assayed by a chemiluminescence (IMMULITE 2000; DPC ‐ Diagnostic Products Corp., Inc., Los Angeles, CA, USA). The intra‐ and interassay coefficients of variation (CV) were 5.8 and 6.0%, respectively. The International Reference Preparation (IRP) for GH was the 98/574. IGF‐I was measured by a chemiluminescence (IMMULITE 2000; DPC). The intra‐ and interassay CV were 3.6 and 6.6%, respectively. The IRP for IGF‐I was the 87/518. The IGF‐I results were expressed as percentage of the upper limit of normal range (%ULNR).

#### Immunohistochemistry

Paraffin‐embedded tissue sections from somatotropinomas (3 μm) were dewaxed, rehydrated and submitted to heat‐mediated antigen retrieval for sst2 and sst5 with Declere^®^ (Cell Marque, Rocklin, CA, USA), pH 6.0 in a cooker pressure for 15 min. Endogenous peroxidase was inhibited in peroxide block (Cell Marque) for 10 min., followed by blocking unspecific immunoglobulin binding with background block (Cell Marque) for 10 min. before primary antibody incubation.

Rabbit monoclonal antibodies directed against sst2 antigen (UMB‐1, 1:5000, Abcam, Cambridge, UK, cat. number ab 134152) and sst5 (UMB‐4, 1:2000, Abcam, cat. number ab 109495) were used. Antibodies were amplified with HiDef Detection^TM^ HRP Polymer System (Cell Marque) and revealed by the chromogen substrate diaminobenzidine (Liquid DAB, Cell Marque). The tissues were counterstained with haematoxylin. Normal human pituitaries obtained from autopsies were used as positive control.

High‐quality images of immunostained tumour cells were randomly captured using a 40× objective lens (ten microscopical fields). Only membrane‐bound positivity was considered for quantification. Immunoreactivity scoring system (IRS) was used to evaluate immunoexpression. The IRS was calculated by the product of the percentage of positive cells (4, >80%; 3, 51–80%; 2, 10–50%; 1, <10%; 0, 0%) and the intensity of the staining (3, strong; 2, moderate; 1, mild; and 0, no staining), ranging between 0 (no staining) and 12 (maximum staining) 16. High IRS was defined as ≥6 17. The samples were analysed by two observers and discordant results submitted to an evaluation by a third observer.

#### Quantitative PCR

The expression of *β‐arrestins 1 and 2*,* sst2, sst5* and *D2* in somatotropinomas was analysed by quantitative real‐time RT‐PCR (qPCR) using Sybergreen^®^ method as previously described 18. The ribonucleic acid (RNA) was isolated using Allprep^®^ Universal kit (Qiagen, Hilden, Germany) and treated with DNase (Turbo DNA‐free™ Kit, Invitrogen, CA, USA), according to the manufacturers’ protocol. The quality and amount of the extracted RNA were evaluated using NanoDrop Lite Spectrophotometer (Thermo Fischer, Wilmington, DE, USA) and Qubit 3.0 Fluorometer (Life Technologies, Foster City, CA, USA), respectively.

The reverse transcription (RT) reaction was performed with 1 μg of total RNA in a final volume of 40 μl containing 10× RT Random Primers, 25× dNTP Mix, 50 U/μl MultiScribeTM Reverse Transcriptase, 20U/μl RNase Inhibitor and 10× RT Buffer [using the first strand cDNA synthesis kit (High‐capacity cDNA reverse transcription kit, Life Technologies)]. The cycling condition was 25°C for 10 min., 37°C for 120 min. and 85°C for 5 min.

GeNorm 3.3 Visual basic application for Microsoft Excel (http://medgen.ugent.be/wjvdesomp/genorm/) was used to evaluate the best housekeeping genes that could be suitable as internal controls in the present cohort, as previously validated by Vandesompele *et al*. 19. Specifically, glyceraldehyde‐3‐phosphate dehydrogenase (GAPDH), β‐actin (ACTB) and hypoxanthine‐guanine phosphoribosyl transferase (HPRT) were found to be stable genes using the qPCR analysis in our cohort of samples.

One μL aliquots of cDNA were amplified by qPCR using the primers specified in Table 1 and the Brilliant III SYBR Green Master Mix in the Stratagene Mx3000p instrument (Agilent, Madrid, Spain). Thermal cycling profile consisted of a pre‐incubation step at 95°C for 3 min., followed by 40 cycles of denaturation (95°C for 20 sec.), annealing and extension (60°C for 20 sec.), and finally, a dissociation cycle to verify that only one product was amplified.

**Table 1:** Primer sequences, product sizes and GenBank accession numbers used for quantitative assessment of β‐arrestin 1, β‐arrestin 2, sst2, sst5 and D2 and housekeeping genes (ACTB, HPRT1, GAPDH) by real‐time RT‐PCR

Gene	Sense	Antisense	Product size (PB)	Genbank accesion no.
ACTB	ACTCTTCCAGCCTTCCTTCCT	CAGTGATCTCCTTCTGCATCCT	176	NM_001101
HPRT1	CTGAGGATTTGGAAAGGGTGT	TAATCCAGCAGGTCAGCAAAG	157	BT019350
GAPDH	AATCCCATCACCATCTTCCA	AAATGAGCCCCAGCCTTC	122	NM_002046
SST2	GGCATGTTTGACTTTGTGGTG	GTCTCATTCAGCCGGGATTT	185	NM_001050
SST5	CTGGTGTTTGCGGGATGTT	GAAGCTCTGGCGGAAGTTGT	183	NM_001053
D2	CGAGCATCCTGAACTTGTGTG	GCGTTATTGAGTCCGAAGAGG	172	NM_016574
ARRB1	CCAACAAGACGGTGAAGAAGA	TCAGTGTGTAGACCTTGCAGAAC	153	NM_004041

ACTB, β‐actin; HPRT, hypoxanthine‐guanine phosphoribosyl transferase; GAPDH, glyceraldehyde‐3‐phosphate dehydrogenase; sst, somatostatin receptor; D2, dopamine receptor; ARRB, β‐arrestin.

Standard curves were constructed using aliquots of purified PCR products serial diluted to obtain standards containing 10^0^, 10^1^, 10^2^, 10^3^, 10^4^, 10^5^ and 10^6^ copies of each transcript analysed per microlitre. As previously described 20, each standard curve was run in parallel with the samples to estimate the mRNA copy number in each sample. The geometric mean of the copy numbers for the three endogenous controls was calculated generating a normalization factor (NF) using the GeNorm 3.3 Visual basic application. Results were then reported as target gene copy number/NF.

#### Statistical analysis

Analyses were performed with SPSS version 23.0 for Mac (SPSS, Inc., Chicago, IL, USA). In the descriptive analysis, categorical variables were expressed through their percentage and frequency and numerical variables were described as median values (minimum–maximum). To compare numeric variables between groups, the Mann–Whitney non‐parametric test was used. To correlate numeric variables, the Spearman test was used. *P* value <0.05 was considered significant.

## Results

### Patient/sample characteristics

Of 96 patients who participated in the study, 44 patients were female (46%). The median age at diagnosis was 43 years (15–75 years). Data of tumour size were available from 72 patients (61 macroadenomas × 11 microadenomas) and of tumour invasiveness from 33 patients (19 non‐invasive × 14 invasive). All invasive tumours were macroadenomas. The median GH at diagnosis was 18.8 μg/l (1.1–120), and IGF‐I at diagnosis was 325%ULNR (101–734; Table 2). None of the patients received radiotherapy before surgery. Only 40 tumour specimens (from the 96 patients included in the study) were available for analysis of sst2 and sst5 by immunohistochemistry.

**Table 2:** Patient characteristics

Patient/sample characteristics	Number of patients	Results
Age	96	43 (15–75)
Female/Male	96	44/52
Macroadenoma/Microadenoma	72	61/11
Invasive (Knosp 3/4)/Non‐invasive	33	14/19
Baseline GH (μg/l)	62	18.8 (1.1–120)
Baseline IGF‐1 (%ULNR)	63	325

%ULNR, percentage of the upper limit of normal range.

In relation to treatment with first‐generation SRL before surgery, 21 patients did not have available data, 62 patients were treatment naïve and 13 patients were treated before surgery. Of the latter, two patients also used cabergoline.

Data regarding response to first‐generation SRL were available in 40 and from these 23 (57.5%) were controlled. Of these, one patient was excluded of the *sst2* and *sst5* analyses because we did not have qPCR data available (probably due to the poor quality of the sample obtained).

### *β‐arrestins, sst2, sst5 and D2* mRNA expression levels

The median *β‐arrestin 1*,* β‐arrestin* 2, *sst2*,* sst5* and *D2* mRNA copy numbers were 478; 9375; 731; 156; and 3989, respectively (Table 3; Table S1).

**Table 3:** Median mRNA expression levels of β‐arrestin 1, β‐arrestin 2, sst2, sst5 and D2

Gene	Number of copies median	Number of copies range, min–max
β‐arrestin 1	478	57–5946
β‐arrestin 2	9375	2701–75,386
Sst2	731	12–23,954
Sst5	156	0–5555
D2	3989	43–40,245

Expression levels are normalized against three housekeeping genes (glyceraldehyde‐3‐phosphate dehydrogenase—GAPDH, β‐actin—ACTB and hypoxanthine‐guanine phosphoribosyl transferase—HPRT). sst: somatostatin receptor, D2: dopamine receptor.

The median *sst2* mRNA levels was lower in patients who used SRL before surgery (*n *=* *13) compared to those who did not (*n *=* *62) [96 (12–23,954) × 937 (34–13,995), respectively, *P *=* *0.001], but we did not find difference in relation to *β‐arrestin 1* and *2*,* sst5* or *D2* mRNA expression in patients who were treated or not before surgery.

### *sst2* and *sst5* protein expression

sst2 was expressed in 97.5% of the patients (39/40), and sst5 in 95% of the patients (38/40) with median scores of 12 (0–12) and 6 (0–12), respectively. High expression, as previously described in the methods section, of sst2 and sst5 was found in 87.5% (*n *=* *35) and 65% (*n *=* *26) of patients, respectively (Table S1).

### Correlation between *β‐arrestins 1* and *2* with, *sst2, sst5, D2* mRNA expression levels and sst2, sst5 protein levels

We found a clear positive correlation between *β‐arrestins 1* and *2* (*R *=* *0.444, *P *<* *0.001). However, no correlation was found between *β‐arrestins 1 or 2* and *sst2*,* sst5* or *D2* mRNA levels or with the protein levels of and sst2 or sst5.

There was no significant correlation between *β‐arrestin 1* and *β‐arrestin 2* with *sst2, sst5* and *D2* mRNA levels in patients who were treated before surgery or in those treatment naïve.

### Relation of *β‐arrestins 1* and *2* with the response to first‐generation SRL and with tumour invasiveness

There was no difference between mRNA levels of *β‐arrestin 1* [592 (122–4642) *versus* 559 (137–5763), *P *=* *0.787] and *β‐arrestin 2* [7960 (5566–43,024) *versus* 9394 (2701–57,088), *P *=* *0.787] in patients who achieved or not biochemical control, respectively. In addition, there was no difference between the mRNA levels of *β‐arrestin 1* [426 (168–2212) *versus* 425 (194–2352) *P *=* *0.900] and *β‐arrestin 2* [8173 (5411–49,863) *versus* 9506 (6868–73,369), *P *=* *0.321] in invasive and non‐invasive tumours, respectively. Notably, *sst2* mRNA levels were higher [1204 (92–23,954) *versus* 231 (12–13,995), *P *=* *0.001] in patients who achieved biochemical control compared to uncontrolled patients. In contrast, there was no difference in *sst5* (*P *=* *0.726) or *D2* (*P *=* *0.279) mRNA levels between controlled and uncontrolled patients with SRL treatment.

Given that *sst2* mRNA levels were found to be lower in patients who used SRL before surgery compared to those who did not, we implemented a separated analysis in the case of *sst2*. Specifically, fifteen of the 40 patients with available data regarding the response to first‐generation SRL treatment were excluded from this analysis because they did not have available data on the use of SRL before surgery (*n *=* *5) or in the *sst2* mRNA levels (*n *=* *1), or because they had been pre‐treated with SRL before surgery (*n *=* *9). Of the available 25 patients, 13 (52%) were controlled. In these patients, the *sst2* mRNA expression was also higher in patients who achieved biochemical control *versus*. uncontrolled patients [1204 (254–23,954) *versus* 478 (12–13,747), *P *=* *0.04]. On the other hand, *β‐arrestins* mRNA levels were similar between controlled and uncontrolled patients.

## Discussion

β‐arrestins are multifunctional adaptor molecules and they have several binding partners. They bind to various GPCRs and mediate their internalization 9, having a role in endocytosis and desensitization of sst2 10. Moreover, β‐arrestins seem to be involved in regulation of cell proliferation 12. Therefore, based on this information, it has been previously suggested that β‐arrestins might be associated with the responsiveness to first‐generation SRL in patients with acromegaly 21. In the present study, we correlated the expression levels of β‐arrestins with sst2, sst5 (at the mRNA and protein levels) or D2 expression and investigated the association of β‐arrestins expression with SRL responsiveness and tumour invasiveness in somatotropinomas.

We found low *β‐arrestin 1* mRNA levels compared to the expression of *β‐arrestin 2* in somatotropinomas, which is in agreement with previous studies 22, 23, 24. Specifically, these low expression levels were also observed in normal rat pituitary tissue and in the rat GH3 cell line 25. In this sense, our study indicates that the use of first‐generation SRL before surgery did not influence *β‐arrestins 1* or *2* mRNA expression in agreement with the data reported by Gatto *et al*. 24.

Interestingly, we found clear positive correlation between the expression levels of *β‐arrestins 1* and *2* in somatotropinomas which might have an important clinical implication as both *β‐arrestins* are able to bind to GPCR 9. In fact, β‐arrestins have been shown to affect the rate of sst2 and sst5 internalization/recycling and desensitization 26, 27, 28. However, we did not find any significant correlation between β‐arrestins 1 and *2* with sst2, sst5 (mRNA and protein levels) or D2 levels, which is also in contrast with a previous study showing an association between *β‐arrestin 1* and *2* mRNA levels with sst2 protein expression 21.

Notably, our study revealed that no correlation exists between *β‐arrestins 1* or *2* mRNA levels and the response to first‐generation SRL treatment in somatotropinomas. In contrast, previous studies by Gatto *et al*. found a negative correlation of *β‐arrestin 1* with response to acute octreotide test *in vivo* and of *β‐arrestin 1* and *β‐arrestin 2* with long‐term treatment with first‐generation SRL 21, 24. Moreover, they found a negative correlation of *β‐arrestin 1* with octreotide treatment *in vitro* 24. In fact, it has been suggested in this report that lower levels of β‐arrestins could lead to a greater number of biologically active receptors on the cell membrane and consequently better response to treatment. Many reasons may explain the discrepancy between this previous study and our present study. Specifically, one of the reasons could be the different criteria to define response to SRL. Gatto *et al*. 21 defined biochemical control with GH levels lower than 2.5 μg/l and age‐matched IGF‐I levels below the ULNR, while we used a different cut‐off point for GH (lower than 1.0 μg/l). Another possible reason is that Gatto *et al*. 21 considered at least 3 months as the minimum time of treatment to define response to SRL, while we believe that a longer duration of treatment is necessary to better define response to treatment and that is the reason why we defined treatment outcome after 6 months of therapy. In addition, another reason for such difference could be based on the methodology used, in that the qPCR analyses used in our study included three housekeeping genes as internal controls, while Gatto *et al*. 21 used only one housekeeping gene which might alter the relative expression levels of the transcripts analysed across the samples as no unequivocal single reference gene (with proven invariable expression between cells/tissue samples) has been identified yet 29. Therefore, as the best alternative, the mean expression of multiple housekeeping genes is recommended to be used for normalization of tumoral samples 30. Furthermore, it should be mentioned that the absence of *β‐arrestins* correlation with the long‐term response to first‐generation SRL in patients with somatotropinomas observed in the present study is in agreement with the clinical data showing an absence of tachyphylaxis during this treatment 4. However, single‐centre studies in acromegaly, as ours, usually have a small number of patients and, therefore, a type II statistical error cannot be excluded. In addition, Gatto *et al*. 21 found that *sst2*/β*‐arrestin 1* and *sst2*/β‐*arrestin 2* ratios seem to be a better predictor in response to first‐generation SRL compared to *sst2* mRNA analysis alone. However, in our case, the relationship did not prove to be a better predictor compared to *sst2* alone (data not shown). Therefore, as these are the only two studies in the literature evaluating the influence of the β‐arrestins in the response to long‐term first‐generation SRL treatment, additional studies are necessary to clarify this issue.

Previous studies had shown involvement of both β‐arrestins at cell cycle regulation, cell migration and apoptotic signalling in other tumours 12, and changes in β‐arrestins expression were associated with more aggressive cancer phenotypes 12, 13, 14. To the best of our knowledge, this is the first study that evaluated the relation between *β‐arrestin 1* or *2* mRNA expression and tumour invasiveness in somatotropinomas. However, different from what has been found in other tumour types 12, 31, 32, we did not find such a relation in somatotropinomas.

## Conclusion

We did not observe a correlation of *β‐arrestins* with *sst2*,* sst5*,* DR2* expression nor a relation of its expression with the response to first‐generation SRL long‐term treatment or tumour invasiveness in this subset of patients with acromegaly. Considering the divergence of our data with previous data in the literature, further studies are necessary to clarify whether β‐arrestins have a role in predicting the response to first‐generation SRL treatment in patients with acromegaly.

## Disclosure

MRG has received unrestricted research grants and lecture fees from Novartis, Ipsen and Pfizer, has participated on advisory boards of Novartis and Ionis and is PI in clinical trials by Novartis and Ipsen. The other authors have nothing to disclosure.

## Supporting information


**Table S1** *β‐arrestin 1*,* β‐arrestin 2*,* sst2*,* sst5*,* D2* mRNA levels in individual somatotropinomas estimated mRNA copy number corrected by a normalization factor (NF) derived from the expression of three housekeeping genes (glyceraldehyde‐3‐phosphate dehydrogenase ‐ GAPDH, β‐actin ‐ ACTB and hypoxanthine‐guanine phosphoribosyl transferase – HPRT) together with sst2 and sst5 protein evaluation (as IRS)
