# Supplementary material for: Molecular evidence and clinical importance of β‐arrestins expression in patients with acromegaly
Source: J Cell Mol Med. 2018 Jan 27;22(4):2110–6. doi: 10.1111/jcmm.13427 (PMC5867117; doi:10.1111/jcmm.13427)
Supplement: Supplementary file 1 — Table S1 β‐arrestin 1, β‐arrestin 2, sst2, sst5, D2 mRNA levels in individual somatotropinomas estimated mRNA copy number corrected by a normalization factor (NF) derived from the expression of three housekeeping genes (glyceraldehyde‐3‐phosphate dehydrogenase ‐ GAPDH, β‐actin ‐ ACTB and hypoxanthine‐guanine phosphoribosyl transferase – HPRT) together with sst2 and sst5 protein evaluation (as IRS) [file JCMM-22-2110-s001.docx]

**Supplementary Table 1.** *β-arrestin 1*, *β-arrestin 2*, *sst2*, *sst5*, *D2* mRNA levels in individual somatotropinomas estimated mRNA copy number corrected by a normalization factor (NF) derived from the expression of three housekeeping genes (glyceraldehyde-3-phosphate dehydrogenase - GAPDH, β-actin - ACTB and hypoxanthine-guanine phosphoribosyl transferase – HPRT) together with sst2 and sst5 protein evaluation (as IRS).

| No. | ARRB1/NF | ARRB2/NF | sst2/NF | sst5/NF | D2/NF | NF | sst2 IRS | sst5 IRS |
| --- | --- | --- | --- | --- | --- | --- | --- | --- |
| 1 | 429 | 6875 | 113 | 60 | 2158 | 2.86 |  |  |
| 2 | 287 | 7348 | 1204 | 120 | 6048 | 1.15 | 12 | 6 |
| 3 | 1622 | 9755 | 1731 | 164 | 3370 | 0.94 |  |  |
| 4 | 954 | 6334 | 255 | 6 | 855 | 2.79 |  |  |
| 5 | 917 | 27687 | 670 | 40 | 1112 | 0.78 | 12 | 9 |
| 6 | 425 | 9506 | 577 | 10 | 12529 | 2.99 | 12 | 1 |
| 7 | 239 | 5841 | 1110 | 103 | 4527 | 2.39 |  |  |
| 8 | 699 | 49863 | 541 | 38 | 601 | 0.06 | 8 | 6 |
| 9 | 312 | 9065 | 1505 | 239 | 1064 | 1.08 | 12 | 12 |
| 10 | 251 | 8439 | 69 | 38 | 1123 | 4.12 | 6 | 4 |
| 11 | 420 | 8674 | 164 | 0 | 7438 | 1.53 | 9 | 0 |
| 12 | 2352 | 24938 | 1194 | 309 | 5208 | 1.00 | 12 | 12 |
| 13 | 690 | 10419 | 120 | 197 | 5803 | 0.32 |  |  |
| 14 | 5946 | 17581 | 97 | 81 | 15483 | 0.64 |  |  |
| 15 | 270 | 5582 | 12 | 178 | 1047 | 0.59 | 4 | 12 |
| 16 | 609 | 16366 | 380 | 40 | 5348 | 2.08 | 8 | 4 |
| 17 | 503 | 12394 | 645 | 8 | 7404 | 2.44 | 12 | 2 |
| 18 | 803 | 15267 | 404 | 65 | 10513 | 2.80 | 12 | 9 |
| 19 | 578 | 8407 | 1597 | 160 | 12808 | 2.76 | 12 | 6 |
| 20 | 5763 | 29611 | 241 | 62 | 4959 | 0.81 | 6 | 6 |
| 21 | 265 | 11994 | 906 | 39 | 1952 | 0.68 | 12 | 4 |
| 22 | 523 | 7920 | 63 | 666 | 4519 | 3.75 | 6 | 9 |
| 23 | 950 | 7802 | 236 | 142 | 1467 | 2.26 | 6 | 9 |
| 24 | 486 | 12952 | 166 | 91 | 4393 | 1.42 | 6 | 9 |
| 25 | 303 | 8594 | 492 | 34 | 3179 | 2.65 | 12 | 2 |
| 26 | 284 | 5658 | 2002 | 105 | 76 | 1.44 |  |  |
| 27 | 348 | 7912 | 1701 | 98 | 2472 | 1.05 | 12 | 9 |
| 28 | 451 | 5821 | 1901 | 1076 | 43 | 1.29 | 12 | 12 |
| 29 | 202 | 7436 | 937 | 123 | 1319 | 2.63 |  |  |
| 30 | 725 | 8413 | 892 | 83 | 824 | 2.15 |  |  |
| 31 | 609 | 8425 | 428 | 245 | 6261 | 0.66 |  |  |
| 32 | 401 | 5411 | 96 | 5 | 481 | 1.29 | 12 | 2 |
| 33 | 284 | 7928 | 401 | 125 | 11652 | 1.65 |  |  |
| 34 | 757 | 57088 | 792 | 1319 | 7254 | 0.03 | 12 | 12 |
| 35 | 261 | 14120 | 79 | 189 | 2997 | 4.08 | 4 | 12 |
| 36 | 356 | 10456 | 99 | 15 | 7682 | 2.44 | 4 | 6 |
| 37 | 1139 | 21346 | 3691 | 136 | 1451 | 1.25 | 12 | 8 |
| 38 | 445 | 6868 | 1240 | 27 | 2680 | 2.35 | 12 | 4 |
| 39 | 855 | 8504 | 1527 | 98 | 369 | 1.48 | 12 | 6 |
| 40 | 331 | 20881 | 137 | 201 | 1847 | 0.74 | 12 | 12 |
| 41 | 537 | 7960 | 999 | 58 | 15933 | 2.35 | 9 | 2 |
| 42 | 381 | 12999 | 3578 | 80 | 1877 | 0.57 | 12 | 4 |
| 43 | 403 | 10775 | 17 | 244 | 4157 | 3.90 | 0 | 6 |
| 44 | 559 | 8305 | 199 | 46 | 5817 | 2.41 |  |  |
| 45 | 240 | 5326 | 34 | 11 | 1281 | 5.33 |  |  |
| 46 | 470 | 8455 | 597 | 45 | 19781 | 4.72 | 6 | 2 |
| 47 | 577 | 73369 | 637 | 214 | 2938 | 0.01 | 9 | 0 |
| 48 | 1521 | 10644 | 48 | 14 | 7131 | 0.54 | 9 | 4 |
| 49 | 1753 | 16621 | 950 | 70 | 2471 | 0.68 |  |  |
| 50 | 168 | 7383 | 1206 | 13 | 8432 | 1.78 | 9 | 6 |
| 51 | 291 | 10284 | 1371 | 210 | 1382 | 1.16 | 12 | 12 |
| 52 | 330 | 7398 | 1227 | 8 | 6236 | 5.23 |  |  |
| 53 | 2212 | 20809 | 319 | 152 | 1782 | 1.35 | 6 | 6 |
| 54 | 194 | 9762 | 1406 | 5 | 10482 | 1.98 | 12 | 6 |
| 55 | 1500 | 16691 | 795 | 234 | 8188 | 0.19 |  |  |
| 56 | 1633 | 10026 | 86 | 100 | 2336 | 0.26 | 1 | 6 |
| 57 | 1413 | 11760 | 637 | 84 | 1425 | 1.51 |  |  |
| 58 | 529 | 12187 | 817 | 11 | 3541 | 1.03 |  |  |
| 59 | 367 | 9357 | 462 | 27 | 1217 | 1.45 |  |  |
| 60 | 592 | 5566 | 10747 | 1152 | 5762 | 0.17 |  |  |
| 61 | 812 | 34692 | 961 | 3497 |  | 0.05 |  |  |
| 62 | 632 | 21113 | 637 | 2574 | 2017 | 0.47 |  |  |
| 63 | 322 | 16801 | 2006 | 5321 | 40245 | 0.77 |  |  |
| 64 | 637 | 7372 | 452 | 1601 | 5130 | 7.97 |  |  |
| 65 | 4642 | 24512 | 2189 | 3330 | 1567 | 0.06 |  |  |
| 66 | 619 | 33920 |  |  | 2668 | 0.07 |  |  |
| 67 | 455 | 10754 | 5189 | 2826 | 3359 | 0.90 |  |  |
| 68 | 201 | 75386 |  |  | 25349 | 0.06 |  |  |
| 69 | 1314 | 8582 | 92 | 0 | 13789 | 2.16 |  |  |
| 70 | 1079 | 34846 |  |  |  | 0.03 |  |  |
| 71 | 1245 | 18100 |  |  | 18604 | 0.29 |  |  |
| 72 | 829 | 12806 | 1401 | 2900 | 27383 | 0.21 |  |  |
| 73 | 57 | 16606 | 3546 | 2864 | 7791 | 0.09 |  |  |
| 74 | 137 | 2701 | 13995 | 1813 | 11413 | 7.46 |  |  |
| 75 | 298 | 3321 | 3163 | 1046 | 7703 | 8.12 |  |  |
| 76 | 822 | 16401 | 7648 | 5332 | 11604 | 0.33 |  |  |
| 77 | 3051 | 42152 | 4260 | 1235 | 7849 | 0.06 |  |  |
| 78 | 938 | 4069 |  |  | 13234 | 4.83 |  |  |
| 79 | 298 | 6655 | 416 | 3767 | 3439 | 1.07 |  |  |
| 80 | 222 | 9896 | 1225 | 1852 | 3821 | 1.24 |  |  |
| 81 | 731 | 19045 | 220 | 41 | 2951 | 6.89 |  |  |
| 82 | 261 | 6702 | 194 | 1322 | 2210 | 6.19 |  |  |
| 83 | 597 | 6811 | 159 | 2085 | 4382 | 5.98 |  |  |
| 84 | 260 | 6547 |  |  |  | 1.39 |  |  |
| 85 | 553 | 10852 | 321 | 4242 |  | 1.47 |  |  |
| 86 | 624 | 7096 | 1662 | 1067 | 1238 | 0.26 |  |  |
| 87 | 278 | 9394 | 205 | 3150 | 14440 | 6.38 |  |  |
| 88 | 366 | 6385 | 1230 | 1833 | 2390 | 5.76 |  |  |
| 89 | 227 | 6959 | 641 | 2586 | 3807 | 6.60 |  |  |
| 90 | 275 | 5048 | 637 | 2246 | 18036 | 7.81 |  |  |
| 91 | 570 | 6794 | 2625 | 1639 | 5276 | 7.29 |  |  |
| 92 | 454 | 4665 | 864 | 1567 | 3759 | 5.19 |  |  |
| 93 | 284 | 5319 | 801 | 1124 | 190 | 5.76 |  |  |
| 94 | 122 | 5581 | 23954 | 2407 | 17016 | 3.12 |  |  |
| 95 | 237 | 5152 | 3851 | 5555 | 586 | 5.11 |  |  |
| 96 | 190 | 43024 | 5586 | 4704 | 7373 | 0.07 |  |  |
| Median | 478 | 9376 | 731 | 156 | 3989 |  | 12 | 6 |

ARRB1: β-arrestin 1, ARRB2: β-arrestin 2, sst: somatostatin receptor, D2: dopamine receptor, IRS: immunoreactivity scoring system. Blank cells represent unavailable data.
